# Antimicrobial peptide melittin against *Xanthomonas oryzae* pv. *oryzae*, the bacterial leaf blight pathogen in rice

**DOI:** 10.1007/s00253-016-7400-4

**Published:** 2016-03-07

**Authors:** Wei Shi, Caiyun Li, Man Li, Xicui Zong, Dongju Han, Yuqing Chen

**Affiliations:** Jiangsu Province Key Laboratory for Molecular and Medical Biotechnology, Life Sciences College, Nanjing Normal University, Nanjing, 210023 China

**Keywords:** Melittin, *Xanthomonas oryzae* pv. *oryzae*, Rice

## Abstract

*Xanthomonas oryzae* pv. *oryzae* is a destructive bacterial disease of rice, and the development of an environmentally safe bactericide is urgently needed. Antimicrobial peptides, as antibacterial sources, may play important roles in bactericide development. In the present study, we found that the antimicrobial peptide melittin had the desired antibacterial activity against *X. oryzae* pv. *oryzae*. The antibacterial mechanism was investigated by examining its effects on cell membranes, energy metabolism, and nucleic acid, and protein synthesis. The antibacterial effects arose from its ability to interact with the bacterial cell wall and disrupt the cytoplasmic membrane by making holes and channels, resulting in the leakage of the cytoplasmic content. Additionally, melittin is able to permeabilize bacterial membranes and reach the cytoplasm, indicating that there are multiple mechanisms of antimicrobial action. DNA/RNA binding assay suggests that melittin may inhibit macromolecular biosynthesis by binding intracellular targets, such as DNA or RNA, and that those two modes eventually lead to bacterial cell death. Melittin can inhibit *X. oryzae* pv. *oryzae* from spreading, alleviating the disease symptoms, which indicated that melittin may have potential applications in plant protection.

## Introduction

Bacterial leaf blight of rice caused by *Xanthomonas oryzae* pv. *oryzae* is a destructive bacterial disease of rice in growing regions worldwide. As Gram-negative bacterium, this plant pathogen can cause vascular disease by producing yellow green spots on the leaf tips and edges, resulting in gray to white lesions along the leaf veins, which severely reduces the rice quality. The disease incidence ranges from 70 to 80 %, leading to significant crop damage (Basso et al. 2011; Lee et al. 2005). Currently, control relies mainly on chemical pesticides. However, their effects on long-term environmental pollution and carcinogenic effects on humans and other animals limit their future use (Daoubi et al. 2005). Thus, to develop new antimicrobial resources with reduced negative environmental impacts is urgently needed to replace the traditional synthetic chemical pesticides used in plant protection.

Antimicrobial peptides (AMPs) are important host defense molecules involved in innate immunity. To date, almost 2100 peptides with antibacterial activity had been discovered from different species (http://aps.unmc.edu/AP/). They are small (∼10–50 residues), generally amphipathic molecules, and most of them contain cationic and hydrophobic residues in elevated proportions. Natural AMPs exhibited a broad activity to directly kill bacteria, yeasts, fungi, viruses, parasites, and even cancer cells. These activities are diverse, specific to the type of AMPs (Zhang and Gallo 2016). The use of AMPs as novel antibiotics in medical application has been proposed and widely accepted for a long time. Although there are the numerous models to explain their mechanism of action ranging from pore formation to general membrane disruption, in fact, it is a complicated interaction between different AMPs and different microbial membranes, which govern membrane selectivity of AMPs (Lee et al. 2016). Besides the use in medical application, AMPs have possible roles as agriculture pesticides for plant disease control because of their short sequences, broad antimicrobial spectra, and diverse sources (Montesinos 2007). Moreover, their mode of action, mainly targeting the microbial cell membrane directly, is thought to reduce the risk of resistance development in microbial population. AMPs have been reported as candidates for plant protection against bacterial and fungal pathogens. Until now, several natural AMPs, such as cecropin (silkmoth) and some modified AMPs, were reported in vitro and ex vivo (detected leaves or fruits) against plant pathogens (Alan and Earle 2002; Coca et al. 2006; Zeitler et al. 2013). However, almost no effective AMPs have been reported against *X. oryzae* pv. *oryzae*, the most important bacterial pathogen.

Melittin, the main component of European honeybee venom from *Apis mellifera*, is a cationic peptide (+5 net charge) composed of 26 amino acid residues (GIGAVLKVLTTGLPALISWIKRKRQQ). Melittin has diverse activities, including antibacterial, antifungal, antiviral, anticancer, and anti-inflammatory, as well as wound-healing potential (Alia et al. 2013; Falco et al. 2013; Park and Lee 2010). It has membrane activity as well as the ability to form pores across the lipid bilayer (Lee et al. 2013). Several studies demonstrated that mellitin exhibits a broad-spectrum antibacterial activity and is more active against Gram-positive than Gram-negative bacteria (Al-Ani et al. 2015). A tremendous amount of work has been done on antibacterial activity of melittin against human and animal pathogenic bacteria (Asthana et al. 2004; Liu et al. 2013). However, very little is known about the ability of natural melittin to act against plant pathogens, and specifically *X. oryzae* pv. *oryzae*. The objective of the present study is to determine the antibacterial activity of melittin against *X. oryzae* pv. *oryzae* and assess its protective effect against rice leaf blight.

## Materials and methods

### Bacterial strains, peptide synthesis, and reagents

*X. oryzae* pv. *oryzae* strain ZJ-173 (which is commonly used in China) was used in this study. *X. oryzae* pv. *oryzae* was grown at 28 °C in nutrient broth (NB) medium as described previously(Zhu et al. 2013). Melittin was synthesized using solid-phase methodology at GL Biochemistry Corporation (Shanghai, China). Preparative reverse phase high-performance liquid chromatography (RP-HPLC) resulted in final products deemed >95 % pure. Selective N-terminal fluorescein labeling of the peptide was performed with fluorescein isothiocyanate (FITC) and deemed >95 % homogeneous. 4,6-diamidino-2-phenylindole (DAPI) was purchased from Sigma-Aldrich (St. Louis, MO, USA). The restriction enzymes and DNA extraction kit were purchased from Takara Bio, Inc. (Shiga, Japan), and the TransZol™ UP Plus RNA Kit was purchased from TransGen Biotech Co., Ltd. (Beijing, China). The T-ATPase (total quantity of adenosine triphosphate in the cell) and protein assay kit were purchased from Jiancheng Bioengineering Institute (Nanjing, China). All other reagents and solvents were made in China and were of analytical grade.

### Antibacterial activity assay

*X. oryzae* pv. *oryzae* was prepared for 24 h in NB medium at 28 °C to achieve an inoculum of approximate mid-log phase (OD_600_ ∼0.5). The antibacterial activity was tested using an agar well diffusion assay and a time-to-kill curve assay. For the former, the samples were placed in the wells of a thin agar plate seeded with *X. oryzae* pv. *oryzae*. Bacterial inhibition zones were detected after incubating at 28 °C for 2 days. For the latter, bacteria cultures were treated with different concentrations of melittin. The half maximal inhibitory concentration (IC50) and microbial growth were assessed by measuring the OD_600_ after incubation for different concentrations (2.5, 5, 7.5, 10, 20 μM) and different hours (2.5, 5, 7.5, and 10 h) at 28 °C as reported elsewhere (Tripathi et al. 2015).

### Determination of the DNA and RNA contents

The determinations of DNA and RNA contents were performed using DAPI. Bacteria were incubated with melittin with final concentration of 10 μM for 2.5, 5, and 7.5 h. Phosphate-buffered saline (PBS) was used as a control. The cells were then collected and diluted with distilled water. A triple volume of DAPI was added to the resuspended bacteria. The cell samples were placed in the dark for 10 min. The DAPI fluorescence of cells was observed using fluorescence spectrometry (364 nm for DNA and 400 nm for RNA). Each experiment was repeated three times.

### Scanning electron microscopy (SEM)

After incubation with 10 μM melittin for 30 min at 28 °C, *X. oryzae* pv. *oryzae* was collected by centrifugation at 10,000×*g* for 10 min. After washing three times with PBS, *X. oryzae* pv. *oryzae* was fixed with 4 % (*v*/*v*) glutaraldehyde in PBS at 4 °C for 3 h. After washing three times with the same buffer, the samples were dehydrated separately for 15 min using a graded series of ethanol solutions (50, 70, 80, 90, 95, and 100 % (*v*/*v*)). They were then air dried and sputter coated with gold to avoid charging effects in the microscope. Samples were viewed using a scanning electron microscope at 10 KV.

### Transmission electron microscopy (TEM)

After incubation with 10 μM melittin for 30 min at 28 °C, *X. oryzae* pv. *oryzae* was collected by centrifugation at 10,000×*g* for 10 min. After washing three times with PBS, *X. oryzae* pv. *oryzae* was fixed with 4 % (*v*/*v*) glutaraldehyde in PBS at 4 °C for 3 h and then post-fixed with 1 % osmium tetroxide at 4 °C for another 2 h. After washing three times with PBS, samples were dehydrated separately for 15 min using a graded series of acetone solutions (30, 50, 70, 80, 90, and 100 %) and embedded in resin. The samples were cut into semi-thin sections, prepared on copper grids, and stained with uranyl acetate and lead citrate. Samples were viewed using a transmission electron microscopy system.

### Determination of intracellular ATP depletion

*X. oryzae* pv. *oryzae* was incubated with melittin (10, 20 μM) for 30 min at 28 °C, with PBS as a control. Then, 1 ml of each culture was centrifuged at 12,000×*g* for 10 min and resuspended in 200 μl 0.9 % NaCl solution. The bacteria were disrupted by sonication, and Coomassie brilliant blue R-250 (Beijing Dingguo Biotech Co. Ltd. China) was used to determine the protein content. The T-ATPase level was determined using a commercial assay kit according to the manufacturer’s recommendations. T-ATPase concentrations were expressed in U/mg protein.

### Confocal laser scanning microscopy

*X. oryzae* pv. *oryzae* was incubated with FITC-labeled melittin (10 μM) for 30 min in the dark at 28 °C, with PBS treatment as a control. Then, the samples were centrifuged at 5000×*g* for 5 min. The bacterial pellets were washed three times with PBS. Images were collected using a confocal laser scanning microscope (excitation, 488 nm; emission, 522 nm for the FITC signal).

### DNA/RNA gel retardation assay

The DNA of *X. oryzae* pv. *oryzae* was purified using a DNA extraction kit (TransGen Biotech, Beijing). Total RNA was prepared using the TransZol UP Plus RNA Kit (TransGen Biotech, Beijing) and resuspended in diethyl pyrocarbonate (DEPC)-treated water. Gel retardation experiments were performed as described by Park et al. (1998). Briefly, mixed 200 ng of DNA with different amounts of melittin (10, 100, 200, 400, 600 ng) in 20 μl of binding buffer (5 % glycerol, 10 mM Tris-HCl (pH 8.0), 1 mM EDTA (ethylenediaminetetraacetic acid), 1 mM DL-dithiothreitol, 20 mM KCl, and 50 μg/ml albumin). For RNA binding, an assay was conducted by mixing 300 ng of RNA with melittin (180, 360, 900 ng) in 30 μl of binding buffer. The reaction mixtures were incubated for 1 h at room temperature and then subjected to gel electrophoresis on a 1 % agarose gel. In addition, samples with peptide/DNA weight ratios of 0.5 were dissolved in 0.5 μl Tris-HCl and then digested with 1 μl *Hin*dIII, *Kpn*I, *SacI*, or *Eco*RI*-*HF (Takara Biotech). After incubating at 37 °C for 3 h, the samples were loaded on to a 1 % agarose gel.

### Sodium dodecyl sulfate polyacrylamide gel electrophoresis (SDS-PAGE) analysis

*X. oryzae* pv. *oryzae* were prepared in NB medium at 28 °C to achieve an inoculum of approximate mid-log phase (OD_600_ ∼0.5). Then, melittin, at final concentrations of 5 and 10 μM, was added and cultured in a rotary shaker (220 rpm) at 28 °C. After 5 h, cells were collected and then an SDS-PAGE analysis was performed using 12 % polyacrylamide gels. Gels were stained with Coomassie brilliant blue R-250.

### Disease protection studies

The seeds from the next generation of LYP9 rice, paternal line 9311 (*Oryza sativa* L. ssp. *indica*) were sprouted after sterilization in distilled water at 28 °C for 3 days, then planted into Kimura B nutrient solution (Ma et al. 2001), and trained to the three- or four-leaf stage in an illuminated incubator. The plants were treated with 50–100 μl (1 mM/ml) melittin by injecting the leaf stem or spraying and then placed in a 28 °C light incubator for one night. Rice leaves were inoculated using the scissor-clipping method (Burdman et al. 2004) with a bacterial suspension having an OD_600_ = 0.6. Plants without melittin and bacterial treatments were used as controls. Average lengths were measured 1 week after *X. oryzae* pv. *oryzae* inoculation. The experiment was performed in three replicates.

### Statistical analysis

All experiments were repeated at least three times. Results are expressed as mean values ± standard errors (mean ± SE). The significance of the differences between the treatments and the respective controls were determined based on Student’s *t* test. A level of *P* < 0.05 was considered to be significant.

## Results

### Melittin showed antibacterial activity against *X. oryzae* pv. *oryzae*

**Fig. 1:**
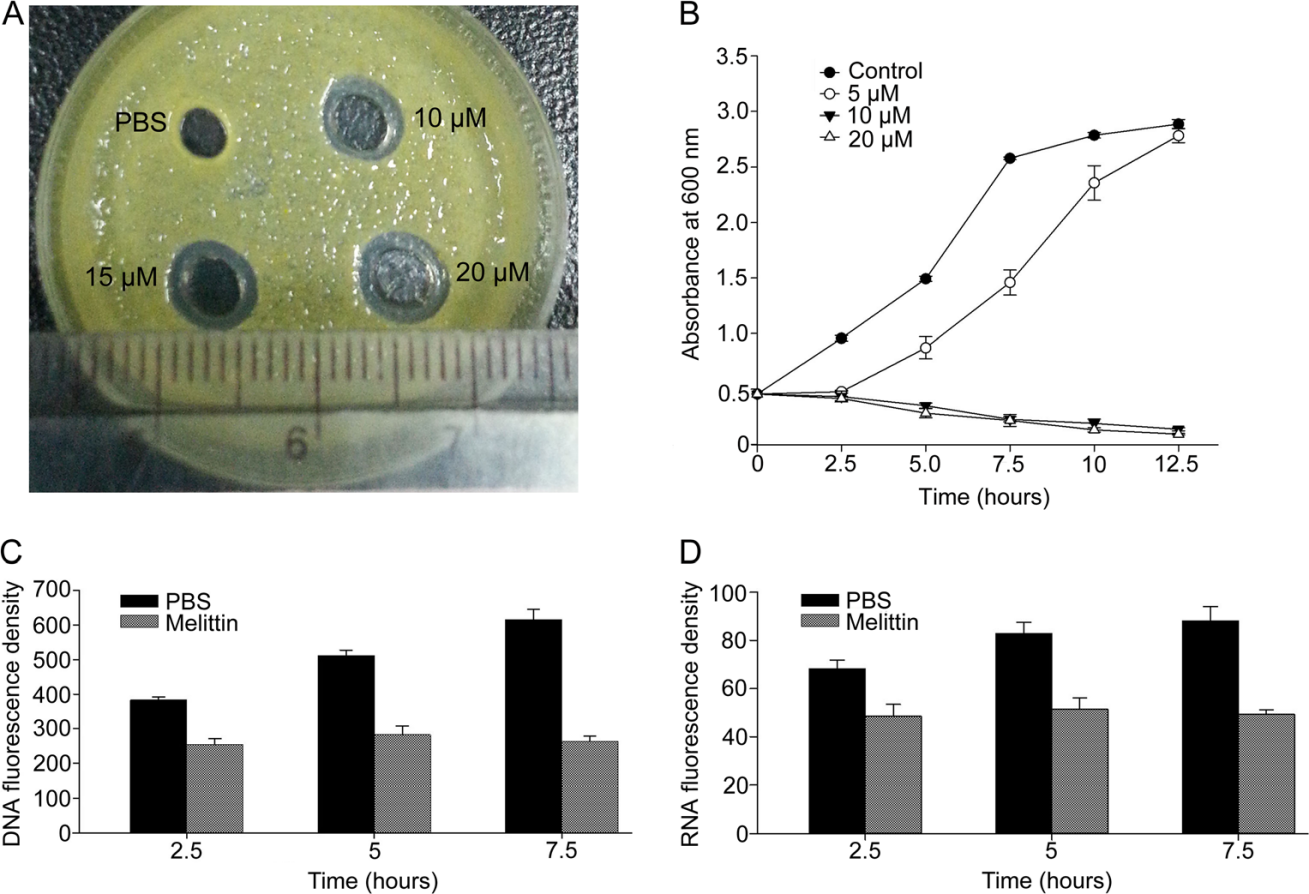
Antibacterial activity of melittin against *X. oryzae* pv. *oryzae*. Agar well assay (**a**), time-to-kill curve assay (**b**), and the determination of DNA (**c**) and RNA (**d**) contents were conducted to detect antibacterial activity

An inhibition zone assay was conducted to detect whether melittin showed antibacterial activity against *X. oryzae* pv. *oryzae* (Fig. 1a). Results showed that melittin has antibacterial activity to the plant pathogen at a concentration of 10 μM. The half maximal inhibitory concentration (IC_50_) determined for melittin was about 9–10 μM. The antibacterial activity was further detected by the time-kill assay (Fig. 1b). At a concentration of 5 μM, melittin inhibited the growth of *X. oryzae* pv. *oryzae* slightly. However, when the concentration increased to 10 μM, the number of viable cells decreased greatly, indicating that the growth of *X. oryzae* pv. *oryzae* was significantly inhibited. After a 10 h incubation, the number of viable cells decreased to an almost undetectable level. The effect of melittin on the nucleic acid content was determined by DAPI staining and fluorescence observations (Fig. 1c, 1d). After treatment with 10 μM melittin, the fluorescence densities of DNA and RNA were reduced significantly, compared with the PBS controls, indicating that the bacterial number decreased greatly or that nucleic acid synthesis was inhibited. All of the data showed that melittin had antibacterial activity against the *X. oryzae* pv. *oryzae* plant pathogen.

### Melittin had the ability to disrupt bacterial cell membrane integrity

**Fig. 2:**
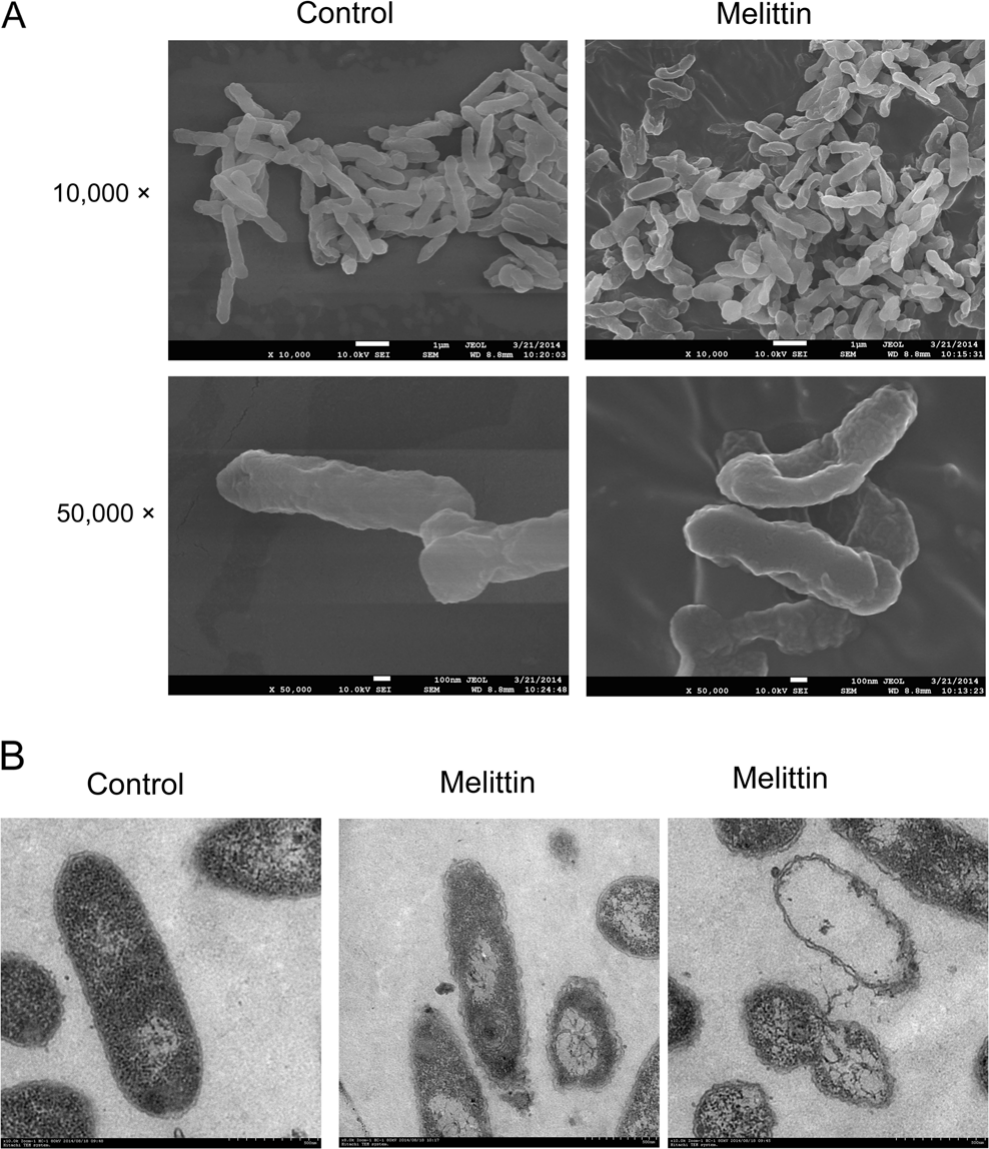
Electron microscopy images of *X. oryzae* pv. *oryzae* treated with melittin. Bacteria were incubated with melittin for 30 min at 28 °C and then observed by scanning electron microscopy (**a**) and transmission electron microscopy (**b**)

The effect of melittin on the membrane of *X. oryzae* pv. *oryzae* was detected using SEM (Fig. 2a). The untreated *X. oryzae* pv. *oryzae* displayed short rods, and a normal smooth and bright surface without any apparent cellular debris. After treatment with 10 μM melittin, obvious cell surface disruption with wrinkles was observed. The bacterial cell membranes were heavily disrupted, which was evident from the formation of potholes on the surface, and more frequent debris, indicating that most of the serious structural changes were caused by melittin. TEM observations indicated that the untreated pathogens were completely filled, having intact bacterial walls and well-defined membranes (Fig. 2b). After treatment with 10 μM melittin, structure changes were observed. There were trumpet-shaped gaps at the end of the bacteria, and there were the regions where the antimicrobial peptide effected the formation of microtubule channels. There was leakage of the bacterial content as a result of wall disruption. The obvious release of the cytoplasmic contents through the membrane was also observed, and there were empty vesicles, which maintained bacterial cell appearance prior to their collapse.

### Melittin had the ability to penetrate the bacterial cell membrane

**Fig. 3:**
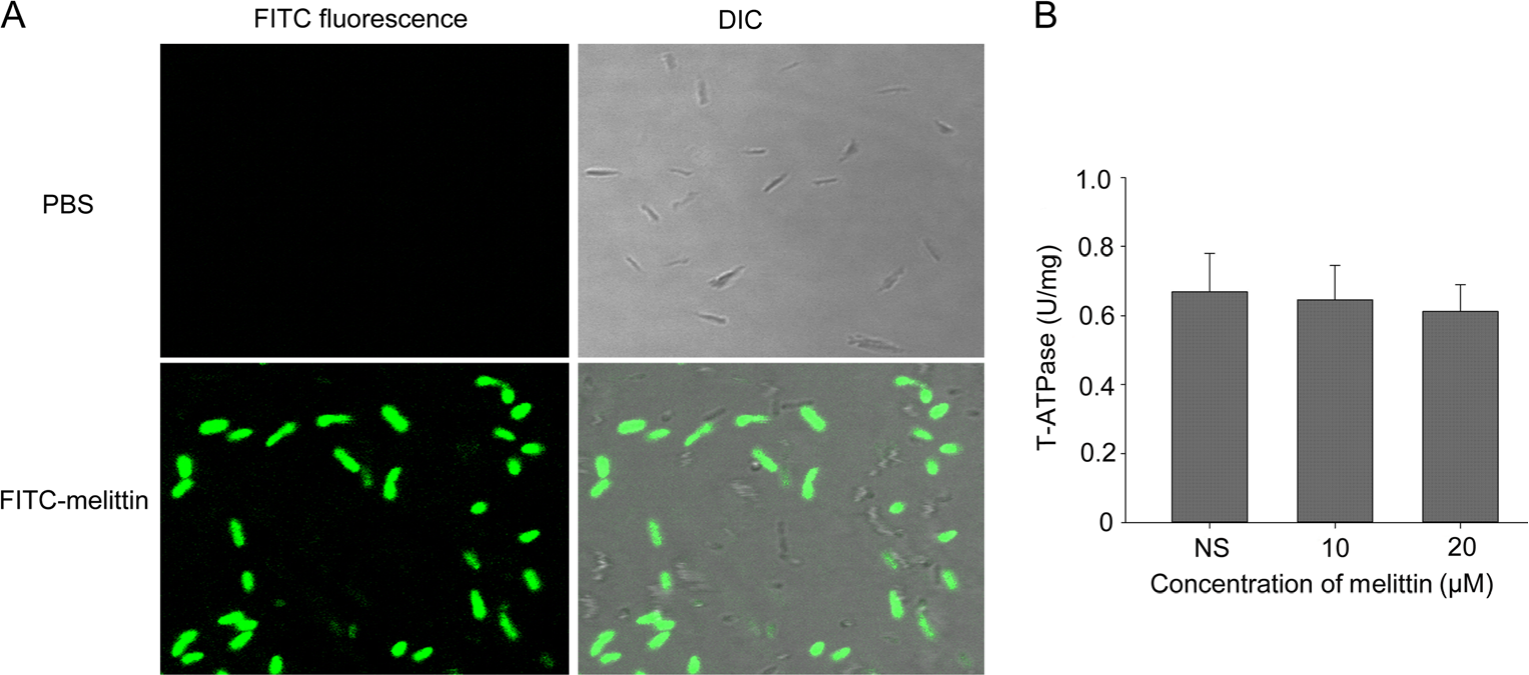
Cellular uptake analyzed by confocal microscopic images. Bacteria were treated with FITC-melittin at a concentration of 10 μM at 28 °C for 30 min (**a**). The effect on energy metabolism was also assessed by determining the ATP consumption (**b**)

FITC-labeled melittin, visualized using a confocal laser scanning microscope, was used to determine whether melittin had the ability to penetrate the bacterial cell membrane. As shown in Fig. 3a, FITC fluorescence accumulated in the cytoplasm of *X. oryzae* pv. *oryzae* after treatment with FITC-labeled melittin for 30 min. Furthermore, FITC fluorescence was mainly amassed at the end of the rod shape. These results indicated that FITC-melittin may penetrate the cell membrane and be distributed in the cytoplasm.

The activity of T-ATPase was further detected to determine whether energy metabolism was affected by melittin. As shown in Fig. 3b, the melittin did not significantly suppress the T-ATPase activity compared with the control. Thus, melittin may have no effect on the energy metabolism of the cells.

### Melittin had binding activity to *X. oryzae* pv. *oryzae* DNA in vitro

**Fig. 4:**
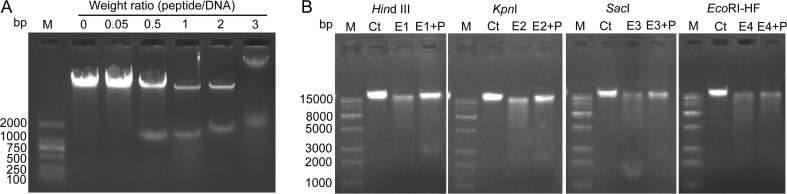
The effect of melittin on the genomic DNA of *X. oryzae* pv. *oryzae*. A gel retardation assay was conducted after various amounts of peptides were incubated with 600 ng of DNA for 1 h. The weight ratios (peptide:DNA) were 0.05, 0.5, 1, 2, and 3 (**a**). Effect of melittin on DNA restriction enzyme digestion by *Hin*dIII (E1), *Kpn*I (E2), *Sac*I (E3), and *Eco*RI*-*HF (E4). *M* marker, *P* peptide melittin (**b**)

A gel retardation assay was conducted to determine whether melittin had DNA-binding activity. As shown in Fig. 4a, at a peptide/DNA weight ratio of 0.05, almost all of the DNA remained at the origin. When the peptide/DNA weight ratio was increased to 0.5, additional *X. oryzae* pv. *oryzae* genomic DNA was degraded, and a DNA fragment of ∼1200 bp was released from the genomic DNA. At peptide/DNA weight ratios of 1 and 2, similar results were observed. In particular, at the peptide/DNA weight ratio of 3, a complete retardation of the DNA was observed, indicating that the DNA was aggregated by melittin. Therefore, melittin had the ability to bind to genomic DNA in vitro and then degraded the genomic melittin DNA to 1000–2000-bp fragments.

Four DNA restriction enzymes with different sites were used to further identify the binding activity of melittin to DNA. As shown in Fig. 4b, after treatment with *Hin*dIII (A/AGCTT), *Kpn*I (GGTAC/C), *Sac*I (GAGCT/C), and *Eco*RI-HF (G/AATTC), *X. oryzae* pv. *oryzae* genomic DNA was cut into different-sized fragments, exhibited as smear patterns by gel electrophoresis. However, the smear fragments decreased after treatment with melittin. All of the data indicated that melittin could bind to genomic DNA in vitro.

### Melittin had binding activity to *X. oryzae* pv. *oryzae* RNA

**Fig. 5:**
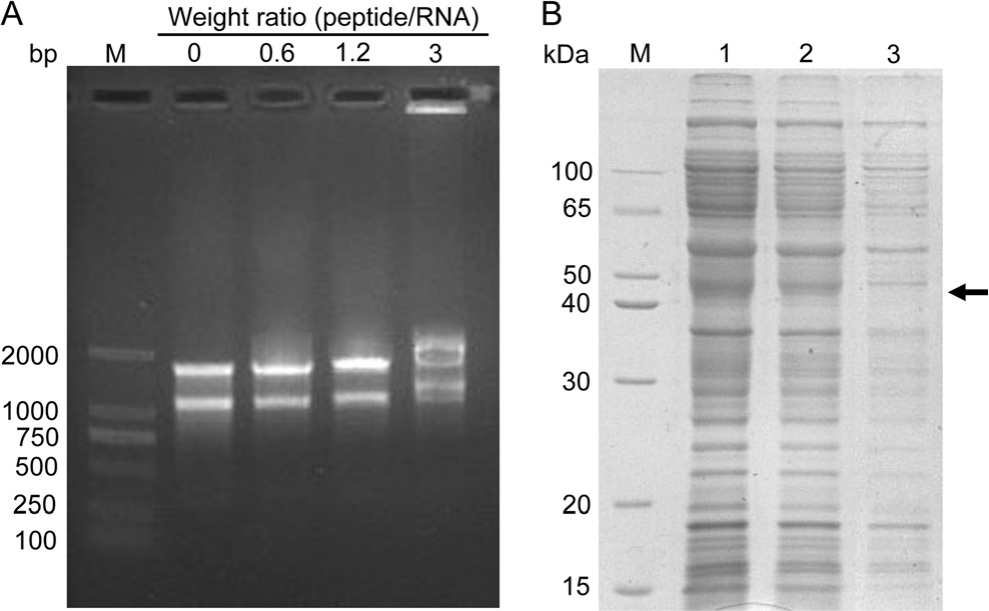
The effect of melittin on RNA. A gel retardation analysis of the binding activity of melittin to RNA. Various amounts of melittin were incubated with 600 ng of RNA for 1 h, the weight ratios (peptide:RNA) were 0.6, 1.2, and 3 (**a**). SDS-PAGE analysis of the change of total proteins in bacteria. *Lane 1*, PBS treatment as a control; *lane 2*, 5 μM melittin treatment for 5 h; *lane 3*, 10 μM melittin treatment for 5 h. The *black arrows* indicate lost protein bands (**b**)

The RNA-binding ability of melittin was evaluated using gel retardation assays. At the peptide/RNA weight ratio of 1.2, the migration of RNA was suppressed by melittin. When the peptide/RNA weight ratio was increased to 3, significant retardation was observed, indicating that melittin had the ability to bind RNA. After treatment with 10 μM melittin for 5 h, the total protein profile of *X. oryzae* pv. *oryzae* was decreased greatly, with the loss of some bands in the SDS-PAGE analysis (Fig. 5b). This result indicated that protein expression may be suppressed by melittin.

### Melittin prevention of *X. oryzae* pv. *oryzae* in rice

**Fig. 6:**
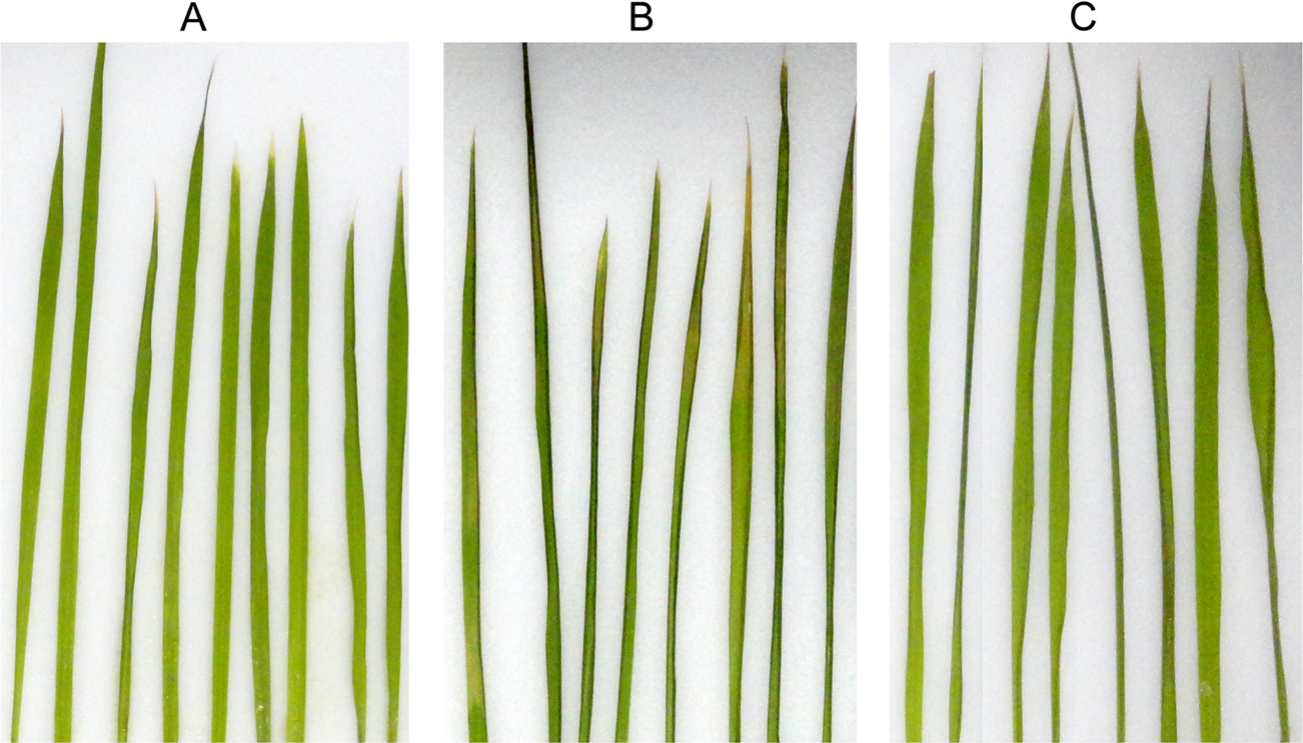
Plant disease reduction by melittin. Normal rice leaves (**a**). Rice leaves infected with *X. oryzae* pv. *oryzae* (**b**). Application of melittin to prevent leaf blight of rice caused by *X. oryzae* pv. *oryzae* **(c)**. The lesions were measured 7 days after inoculation

Rice plants at the three- or four-leaf stage were used to establish a model of the rice bacterial leaf blight by injecting with the *X. oryzae* pv. *oryzae* pathogen. As shown in Fig. 6a, 6b, the development of disease symptoms was predominant in the bacterial blight disease model when compared with the non-injected plants. After treatment with melittin, the development of disease was controlled (Fig. 6c). The results from the lesion measurement experiment showed that very short lesions (average 1.38 ± 0.28 cm) were found in the melittin-treated group compared with the positive control group, which was susceptible to the pathogen and had long lesions (average 13.12 ± 0.89 cm). Hence, the melittin treatment is effective for the protection of rice from the *X. oryzae* pv. *oryzae* pathogen.

## Discussion

Rice is the staple diet of more than three billion people, and the yield must double over the next 40 years if we are to sustain the nutritional needs of an ever-expanding global population (Skamnioti and Gurr 2009). However, rice is vulnerable to disease wherever it is grown and bacterial leaf blight of rice caused by *X. oryzae* pv. *oryzae* is one of the most destructive bacterial diseases worldwide. Che et al. (2011) reported that the Hpa-1-cecropin A (KLFKKIEKV)-melittin (KIFKKIEKKV-AVLKVLTTGL) hybrid peptide inhibited several bacteria and fungi, including *X. oryzae* pv. *oryzae*. However, whether melittin inhibits this plant pathogen is still unknown. In this study, we found that the antimicrobial peptide, melittin, showed antibacterial activity against the *X. oryzae* pv. *oryzae* plant pathogen. Many reports have indicated that melittin possesses broad antimicrobial activities in vitro. We tested the antimicrobial activity of melittin against several plant pathogen, including *Ralstonia solanacearum*, *Magnaporthe grisea*, *Ustilaginoidea oryzae*, *Alternaria alternata* (Fries) Keissler, *Fusarium graminearum* Sehw, and scab of cucurbits. The results showed that melittin had weak antimicrobial activities against these important plant pathogens (data not shown).

A better understanding of the melittin and *X. oryzae* pv. *oryzae* interactions will greatly help develop melittin use in rice plant protection. Previous investigations of the actions of melittin on cell membranes were conducted mainly using synthetic model membranes. They hypothesized that melittin disrupted membrane bilayers via a two-step “detergent-like” mechanism. The first step involves the electrostatic interaction of melittin with negatively charged lipid headgroups. After the concentration of melittin on the lipid surface reaches a critical concentration, melittin rearranges to form pore-like structures that disrupt the membrane bilayer (Lee et al. 2013). However, little research has been conducted using natural membranes of plant pathogens. In our study, FITC-labeled melittin had the binding ability to *X. oryzae* pv. *oryzae*, a Gram-negative plant pathogen, which may have resulted from electrostatic interactions. Melittin also had the ability to cause surface roughening and shrinking, and the formation of potholes, which indicated that the cell membrane structure was disrupted and that channels could be formed by melittin. Moreover, the wall disruption led to the leakage of bacterial contents, with empty vesicles remaining, and debris was also observed, indicating that melittin had the ability to kill cells through membrane-permeability/disrupting mechanism. This resulted in rapid cell death in the *X. oryzae* pv. *oryzae* pathogen. Melittin is a small linear peptide compound of 26 amino acids with a hydrophobic N-terminal region and a hydrophilic C-terminal region. Datiles reported that the ATPase activity of *Escherichia coli* was inhibited by melittin (Datiles et al. 2008). However, melittin had no effect on the energy metabolism of this plant pathogen, as indicated by the activity of the T-ATPase assay.

In addition to membrane-permeabilizing/disrupting properties, many AMPs also interact with intracellular targets or disrupt cellular processes. Our study together with other studies showed that melittin killed bacteria mainly by permeabilizing/disrupting the microbial cytoplasmic membrane (Lee et al. 2013). Very interestingly, using FITC labeling and confocal microscopy, we found that melittin not only bound the plasma membrane, but also entered cells. Thus, melittin used its membrane binding properties to kill bacteria by rapidly lysing the cells. At the same time, melittin also had the chance to enter the cells, which may have resulted from the disruption of the membrane. In eukaryotes, melittin has several mechanisms to kill yeast and cancer cells (Gajski and Garaj-Vrhovac 2013). Park and Lee (2010) speculated that melittin could more easily translocate the plasma membrane and then bind to intracellular molecules, which might trigger apoptosis in *Candida albicans* (Park and Lee 2010). In human leukemic U937 cells, melittin could induce Bcl-2- and caspase-3-dependent apoptosis through the downregulation of Akt phosphorylation (Moon et al. 2008).

The gel retardation assay showed that melittin strongly bound to DNA/RNA in vitro, suggesting the possibility of inhibition of intracellular functions via interference with DNA/RNA functions. The five cationic residues in melittin may enable electrostatic interactions with the negatively charged DNA. In addition to cationic residues, a leucine zipper motif, which is a typical DNA-binding domain with every seventh amino acid being leucine/isoleucine, was identified in melittin (Asthana et al. 2004). In fact, besides melittin, several other AMPs inhibit DNA synthesis by binding, including buforin IIB, indolicidin, and NKLP27, which are known to bind DNA, inhibit DNA synthesis, and induce the filamentation of bacteria (Hsu et al. 2005; Zhang et al. 2014). The inhibition of protein synthesis was also observed in our study, as observed in an SDS-PAGE analysis. We also found that the fluorescence density of nucleic acids were reduced significantly by melittin, which may be the result of the death of *X. oryzae* pv. *oryzae* or the inhibition of proliferation.

Finding an antibacterial activity for melittin in vitro experiments is not predictive of its capacity to protect the rice plant in vivo from *X. oryzae* pv. *oryzae*, because several host components could interfere with the AMP’s activity (Montesinos and Bardaji 2008). Therefore, an inhibition assay was conducted to assess the prevention of infection in rice plants exposed to the *X. oryzae* pv. *oryzae* pathogen. Our data showed that melittin exhibited effective protection of rice against *X. oryzae* pv. *oryzae*. Chemical pesticides have been widely used in the past years and their effects on long-term environmental pollution, and carcinogenic effects on humans and other animals, limit their use (Daoubi et al. 2005). AMPs, as a control source with a potentially reduced negative environmental impact and a broad spectrum of activities, have attracted much attention in plant protection research (Morassutti et al. 2002). The quick kill effect of melittin, which is mainly caused by membrane disruption, makes it difficult for *X. oryzae* pv. *oryzae* to develop resistance to melittin. Although there are several problems for melittin, such as its high hemolysis, a lack of selectivity toxicity and high protease degradation, which limit the use of melittin as an antibacterial agent and anticancer agent to human (Asthana et al. 2004; Dempsey 1990; Oren and Shai 1997), application for plant protection might be possible. However, after treatment of rice with melittin, the plant height, tillering ability, and the leaf color and shape were identical to the PBS control. Furthermore, no lesion mimic were observed in our experiment. All these showed that the rice plants were healthy after the melittin treatment, indicating a lack of undesirable toxic effects on rice. This may result from the differences in cell structure between mammal cells and plant cells. Therefore, melittin represents a promising candidate for further development to protect rice from bacterial leaf blight. Expression of several AMP coding genes in plants has been used to enhance their resistance to bacterial and fungal pathogens (Carmona et al. 1993; Che et al. 2011). Additionally, the transgenic plants showed considerably greater resistance to certain pathogens than wild-type plants (Jan et al. 2010; Lakshman et al. 2013; Nadal et al. 2012). Thus, in addition to being used directly as an agricultural pesticide, melittin may be a candidate in developing transgenic rice with resistance to bacterial leaf blight. However, developing less toxic and more stable compounds requires further research. Structural modifications to reduce the hemolytic activity have been conducted (Pandey et al. 2010). Developing more effective and less toxic derivatives of melittin to protect rice from *X. oryzae* pv. *oryzae* is urgently needed.
